# Poikiloderma with neutropenia: a case report

**DOI:** 10.1186/s13256-025-05027-2

**Published:** 2025-01-15

**Authors:** Jebran Chekr, Jan Andraws, Jubran Elias, Diana Alasmar

**Affiliations:** 1 Faculty of Medicine, Damascus University, Damascus, Syria; 2 Department of Pediatrics, University Children Hospital, Damascus University, Damascus, Syria

**Keywords:** Poikiloderma with neutropenia, *C16orf57*, Syria, Usb1, Gene, Mutation

## Abstract

**Background:**

Poikiloderma with neutropenia is a rare genetic disorder primarily characterized by the presence of poikiloderma and congenital chronic neutropenia. Mutations in the *C16orf57* gene, which encodes the USB1 protein, are implicated as the underlying cause of poikiloderma with neutropenia.

**Case presentation:**

Our patient, an 11-year-old Syrian male child who presented with poikiloderma, palmoplantar keratoderma, pachyonychia, recurrent infections, and neutropenia, is considered to be the first documented case in Syria. Clinical examinations, laboratory tests, radiographic imaging, and genetic analyses have been conducted, with the latter being essential and definitive for diagnosis.

**Conclusion:**

This study aimed to evaluate whether poikiloderma with neutropenia should be considered for differential diagnosis because of its diagnostic complexity, emphasizing the importance of follow-up for the early identification of potential complications.

## Background

Poikiloderma with neutropenia (PN), Clericuzio-type (Online Mendelian Inheritance in Man, OMIM, #604,173), is a rare autosomal recessive genodermatosis initially documented among Navajo Native Americans by Clericuzio [1]. To date, PN has been reported in approximately 100 patients in medical literature [2]. It manifests as poikiloderma (telangiectatic lesions, dyspigmentation, and epidermal atrophy), permanent neutropenia, mainly recurrent infections, short stature, nail abnormalities, and palmoplantar hyperkeratosis [1, 3]. Causative mutations in the *C16orf57* gene, encoding the USB1 protein, underlie PN [3]. Diagnosis relies on classic cutaneous manifestations, chronic neutropenia, and identification of *C16orf57* gene mutations [4]. The disease is not yet curable, and treatment aims to manage symptoms, improve quality of life, and reduce future complications [5]. This article succinctly outlines the clinicopathologic features of the first documented PN case in Syria, emphasizing the exceptional rarity of this condition.

## Case presentation

An 11-year-old male Syrian child was referred to the hematology department of the University Pediatrics Hospital in Damascus in January 2023 because of severe neutropenia in routine laboratory tests. He was born through vaginal delivery with a very low birth weight (less than 1500 g) to parents who were third-degree relatives. The child received complete primary vaccinations following Syria’s National Immunization Program.

The patient’s medical history revealed meningitis at the age of 1 year and recurrent respiratory infections at various intervals. There was no history of psychomotor developmental delay during early childhood. At the age of 10 years, before hospitalization, the child underwent herniorrhaphy and appendectomy. He had a history of taking high-dose systemic corticosteroids to treat his dermatological lesions, which were not properly investigated.

**Fig. 1:**
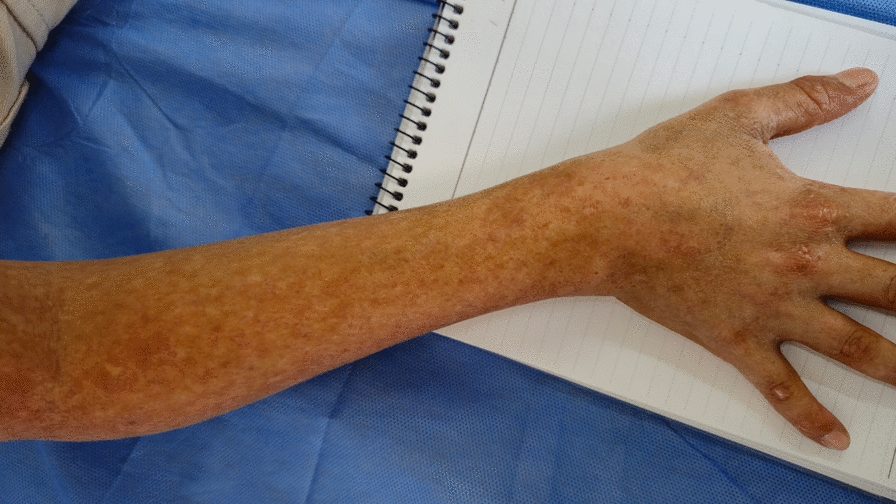
Areas of hypo- and hyperpigmentation distributed along the extensor surface of the upper limb

**Fig. 2:**
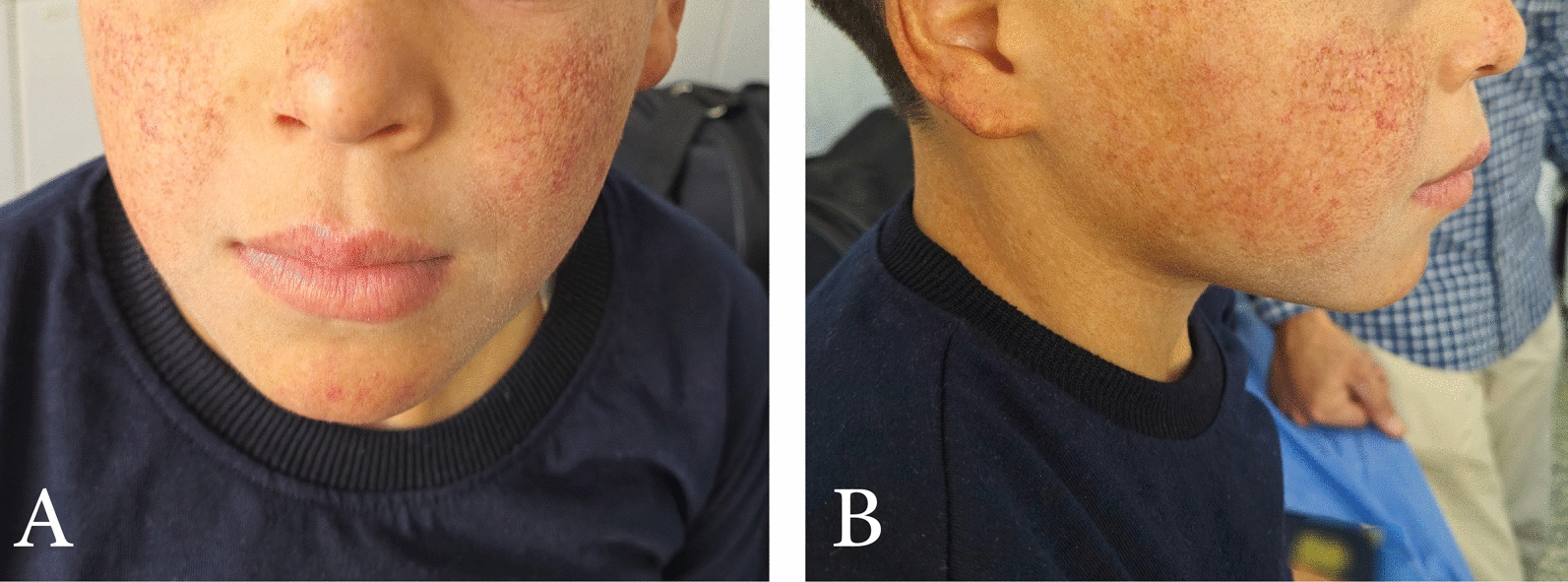
Characteristic dermatological manifestations of Clericuzio-type poikiloderma with neutropenia. **A** Frontal view of the patient’s face. **B** Lateral view of the patient’s face

Upon admission to the hospital and subsequent clinical examination, growth retardation was evident with low height for age (*Z*-score = −2.5). Corticosteroids masked dermatological signs of disease, and upon cessation these signs reappeared, involving areas of hyper and hypopigmentation, atrophy, telangiectasias, and pachyonychia (Figs. 1, 2). Ocular examination and psychomotor development were within normal limits. Physical examination revealed hepatosplenomegaly.

Repeated laboratory investigations yielded consistent results: white blood cell (WBC) count of 2300 cells/μl (neutrophil 437), platelet count of 158,000/µl, lactate dehydrogenase (LDH) level of 608 U/L, testosterone level of 3.82 ng/dL, luteinizing hormone (LH) concentration of 0.26 mIU/ml, and follicle-stimulating hormone (FSH) level of 8.54 mIU/ml. Additionally, erythrocyte sedimentation rate (ESR), C-reactive protein (CRP) level, complement factor (C3 and C4) level, creatine phosphokinase (CPK) level, folate level, and lymphocyte count within normal ranges.

Abdominal ultrasound revealed hepatomegaly (2–3 cm below the costal margin) and splenomegaly (3 cm below the costal margin). Testicular ultrasound revealed atrophy of the left testes and nondescended right testes. Genetic analysis conducted in June 2023 revealed a homozygous nonsense variant mutation (NM_024598.3: c.243G > A; p.[Trp81]) in the *USB1* gene, encoding the U6 small nuclear RNA (snRNA) phosphodiesterase 1, known to cause PN (OMIM#604,173). This variant has previously been reported to be pathogenic.

On the basis of these findings, the patient was diagnosed with poikiloderma with neutropenia.

The incorrect treatment of dermatological manifestations, the patient’s residence in a remote rural area, and the limited availability of genetic diagnostic tools and their high cost all played a significant role in delaying the diagnosis of the condition.

The patient received symptomatic treatment along with recommendations to minimize direct sunlight exposure, screening for skin cancer, and regular pediatric endocrinology evaluation to assess growth and pubertal development. The annual influenza vaccine was given as per the standard of care.

At his most recent follow-up in September 2024, notable improvement in dermatological symptoms was observed, with no new clinical or pathological findings compared with prior evaluations. Additionally, an assessment of psychosocial development revealed no evidence of delay in this aspect.

**Fig. 3:**
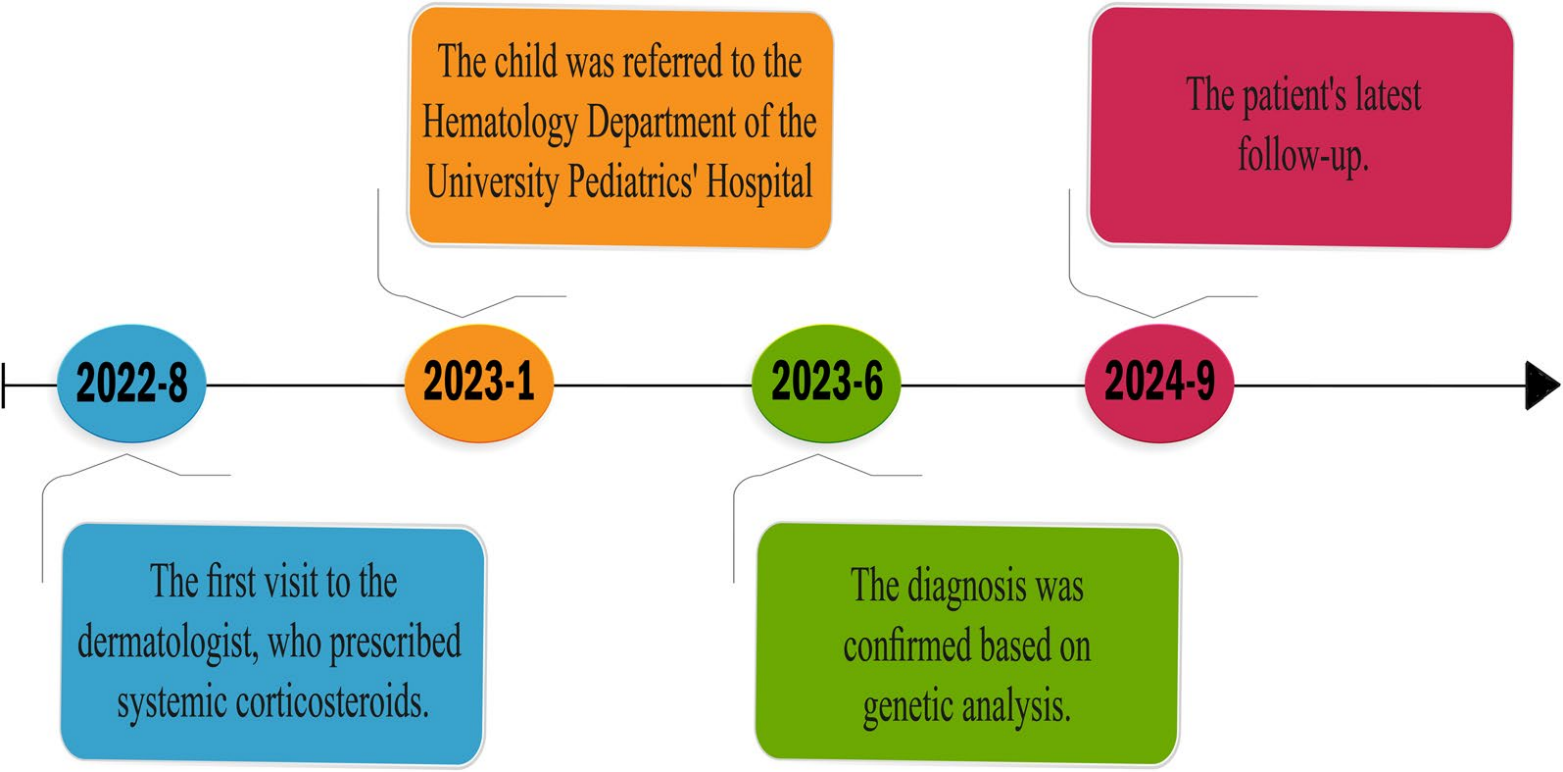
Timeline of events related to this case

A timeline of the patient’s course is presented in Fig. 3.

## Discussion

PN, specifically known as clericuzio-type poikiloderma, is an exceptionally rare autosomal recessive disorder arising from mutations in the *USB1* gene. PN was initially reported by Clericuzio in 1991 among individuals of Navajo Native American descent [6]. It is almost always characterized by the co-occurrence of poikiloderma and neutropenia. The onset of PN occurs in early childhood [7]. PN may present with a wide spectrum of additional manifestations, such as hypogonadotropic hypogonadism, hepatosplenomegaly, and various other atypical features [7]. In this paper, we present the first case of PN diagnosed in Syria out of a few cases reported in medical literature.

PN was clinically diagnosed in our patient and subsequently confirmed through molecular analysis of the *C16orf57* gene [8]. Mutation analysis of the coding region of *C16orf57* revealed a homozygous single nucleotide substitution from guanine to adenine (G to A) at position 243 in exon 2. This transversion results in the conversion of the tryptophan codon (TGG) to a stop codon (TGA), and the resulting nonsense mutation is designated p.W81X [8]. The mutation was confirmed in a heterozygous state in the unaffected parents. The variant c.243G > A, p.(Trp81Ter) identified in our case has previously been documented in at least three families of northern European descent [8–11].

As shown in Table 1, four patients (A, B, C, D) with PN were reported, two of whom (A and B) had the same mutation as our patient, while the other two (C and D) had a different PN mutation.

**Table 1:** Suggested major and minor criteria for the diagnosis of poikiloderma with neutropenia [8]

	Arnold *et al*. [8]	Vincenzo *et al*. [7]	Piard *et al*. [10]	Colombo *et al*. [13]	Current study
	Patient A	Patient B	Patient C	Patient D	Our patient
Patient ID	Patient 1	–	C16-01	#48	Our patient
USB1 genotype	c.[243G > A];[243G > A]	c.[243G > A];[243G > A]	c.[243G > A];c.[267T > A]	c.[243G > A];[541C > T]	c.[243G > A];[243G > A]
Major criteria					
Poikiloderma	+	+	+	+	+
Persistant neutropenia	+	+	+	+	+
Recurrent infections	+	+	−	+	+
Palmoplantar keratoderma	+	+	+	+	+
Pachyonychia	+	+	+	+	+
Photosensitivity	+	+	+	+	−
Minor criteria					
Hepatosplenomegaly	+	+	−	−	+
Nondescended testicles	+	−	NA	−	+
Milia	−	NA	NA	NA	−
Verrucous lesions	−	NA	NA	NA	−
Atrophic scars	−	NA	NA	NA	+
Dental caries	−	+	+	+	+
Lacrimal duct obstruction	−	NA	NA	NA	−
Growth retardation	+	+	−	+	+
Elevated lactate dehydrogenase	+	+	+	−	−
Transient thrombocytopenia	+	−	−	−	+
Transient leukopenia	−	+	+	+	+
Elevated ferritin	+	+	+	−	−
Interface dermatitis	+	NA	NA	NA	−

NA, not available

Our patient met the diagnostic criteria suggested by Arnold *et al*. [8] and has five major features (poikiloderma, persistent neutropenia, palmoplantar keratoderma, and pachyonychia of the hallux nail), along with five minor features (milia, verrucous lesions, elevated LDH, transient leukopenia, and elevated ferritin).

Upon comparing the clinical findings of previously studied cases with our case, we found that the manifestations that are almost always present include poikiloderma and neutropenia. In contrast to the patients mentioned in the table, our patient did not suffer from any symptoms of photosensitivity. Arnold *et al*. [8] and Vincenzo *et al*. [7] described hepatosplenomegaly in their patients, which we also observed in our patient, and this may be attributed to the genetic mutation variants they share. Among other positive findings in our patient were nondescended testicles and transient thrombocytopenia observed in patient A. Additionally, dental caries and transient leukopenia were present in patients B, C, and D. Moreover, we did not find any elevation of ferritin or lactate dehydrogenase in our patient, nor were there any signs of milia or verrucous lesions.

Cutaneous signs were treated with topical corticosteroids. For skin cancer prevention, the application of sunscreen was advised, along with minimizing excessive sun exposure. No granulocyte colony-stimulating factor (G-CSF) was administered, as there is not enough evidence to support this treatment. [5] A prescription for vitamin D (600 IU) per day was given to minimize the potential side effects of prior high-dose systemic corticosteroids. A dental check-up every 3–6 months was recommended [5].

The patient’s prognosis remains uncertain; however, PN, with only a few causative mutations identified to date, appears to predispose individuals to dermatological and hematologic malignancies, including the c.243G > A, p.(Trp81Ter) pathological variant observed in our case [7, 12]. In terms of skin cancer risk, multiple reports have documented the development of squamous cell carcinoma (SCC) in young individuals with PN. Patients face an increased risk of myelodysplastic syndrome and, in rare instances, acute myelogenous leukemia later in adulthood [5, 7, 12]. Therefore, annual assessments of skin cancer and early myelodysplastic changes are recommended for patients [5].

The patient was diagnosed at a late stage owing to the limited availability and high cost of genetic testing, as well as poor financial circumstances. Additionally, there was no initial medical consideration for the syndrome as a differential diagnosis, particularly since this is the first documented case in our country. Therefore, such a diagnosis should be considered when chronic noncyclic neutropenia is associated with poikiloderma to enable early detection and prevent serious complications, especially malignant ones.

This case report may serve as a valuable resource for improving diagnostic skills for rare diseases and supporting ongoing medical education. Furthermore, this type of research lays a foundation for future studies aimed at advancing medical practices both locally and globally.

## Conclusion

In our study, we present the first case of poikiloderma with neutropenia in Syria, thus highlighting the occurrence of this rare disease within our region and emphasizing the importance of its consideration during diagnosis, particularly among patients exhibiting chronic neutropenia concomitant with dermatological manifestations. Moreover, appropriate symptomatic treatment, prophylaxis, and close monitoring for the early detection of complications, especially malignant complications, are crucial.

## Data Availability

Not applicable.

## Abbreviations

PN: Poikiloderma with neutropenia
WBC: White blood cell
LDH: Lactate dehydrogenase
LH: Luteinizing hormone
FSH: Follicle-stimulating hormone
ESR: Erythrocyte sedimentation rate
CRP: C-reactive protein
CPK: Creatine phosphokinase
G: Guanine
A: Adenine
G-CSF: Granulocyte colony-stimulating factor
